# Supplementary material for: A Pilot Study on the Effects of Transcranial Direct Current Stimulation on Brain Rhythms and Entropy during Self-Paced Finger Movement using the Epoc Helmet
Source: Front Hum Neurosci. 2017 Apr 25;11:201. doi: 10.3389/fnhum.2017.00201 (PMC5408787; doi:10.3389/fnhum.2017.00201)
Supplement: Supplementary file 1 [file Data_Sheet_1.zip › Complementary_results/Frequency_Bands_Rest-Referenced_average_PSD_windows/Delta/Delta_Band_Rest-Referenced_avg-win_P8.pdf]

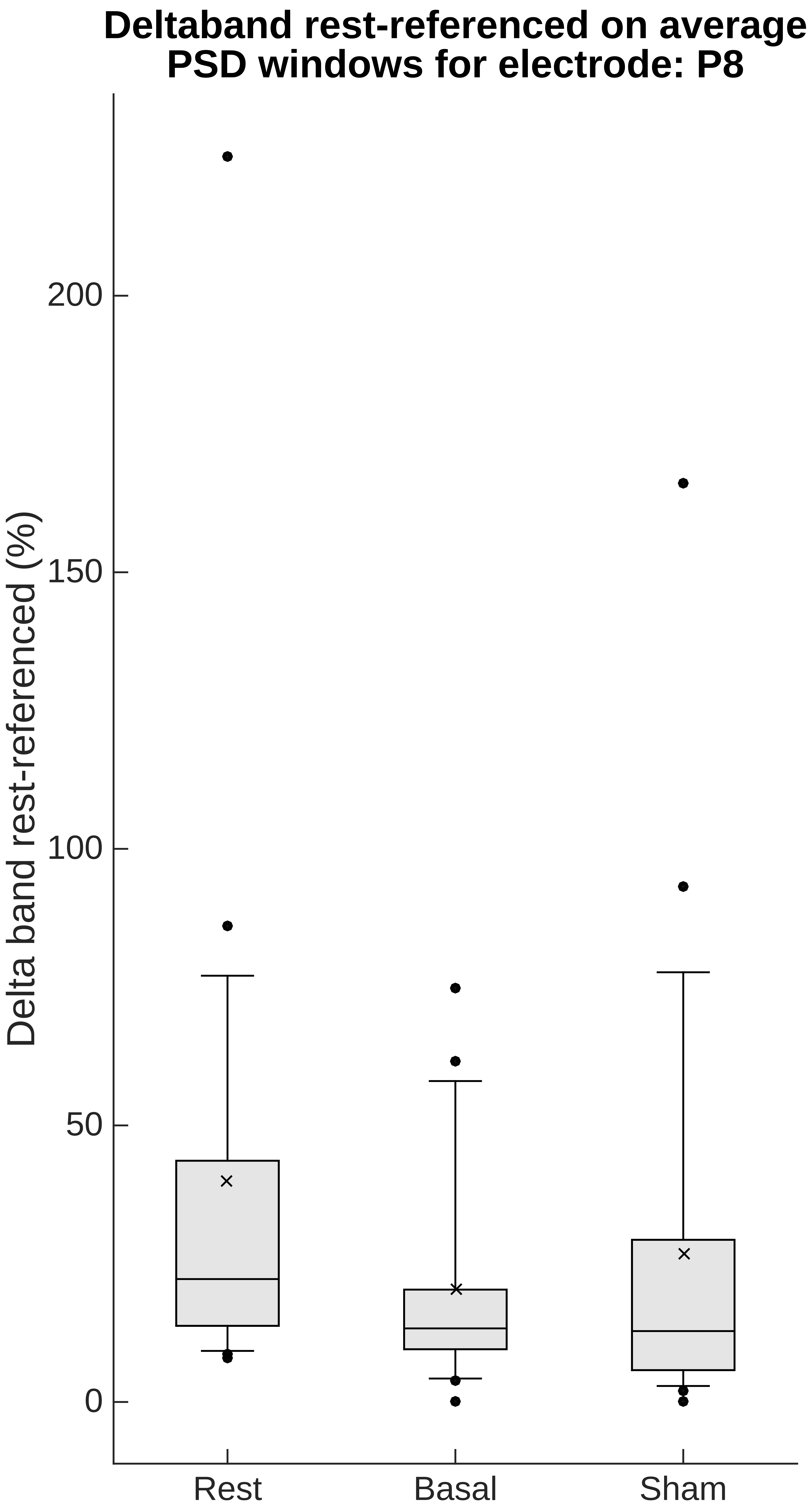

Deltaband rest-referenced on average
PSD windows for electrode: P8
Delta band rest-referenced (%)
200
150
100
50
0
Rest
Basal
Sham
